# A simple and convenient method for the preparation of antioxidant peptides from walnut (*Juglans regia* L.) protein hydrolysates

**DOI:** 10.1186/s13065-016-0184-x

**Published:** 2016-06-21

**Authors:** Ming-Chuan Liu, Sheng-Jie Yang, Da Hong, Jin-Ping Yang, Min Liu, Yun Lin, Chia-Hui Huang, Chao-Jih Wang

**Affiliations:** R&D Center, Sinphar Tian-Li Pharmaceutical Co., Ltd., Hangzhou, 311100 China; School of Life Science and Biopharmaceutics, Shenyang Pharmaceutical Univerisity, Shenyang, 110016 China; R&D Center, Sinphar Pharmaceutical Co., Ltd., Ilan (Taiwan), 269 China

**Keywords:** Large scale preparation, Walnut, Protein, Proteases, Peptide, Antioxidant

## Abstract

**Background:**

Walnut (*Juglans regia* L.), that belongs to the Juglandaceae family, is one of the nuts commonly found in Chinese diets. Researchers had obtained peptides from walnut protein hydrolysates, and these peptides exhibited the high antioxidant activities. The objective of this study was to develop a simple and convenient method for a facile and reproducible preparation of antioxidant peptides from walnut protein hydrolysates.

**Results:**

Walnut proteins were extracted from walnut kernels using continuous countercurrent extraction process, and were separately hydrolyzed with six types of proteases (neutrase, papain, bromelain, alcalase, pepsin, and pancreatin). Then, hydrolysates were purified by ultrafiltration. The yields and purity of the peptides prepared using neutrase and papain were 16 and 81 % at least, respectively, higher than others, and had low molecular weight, 99 % of which were less than 1500 Da. Furthermore, the bioassay indicated that the two peptides exhibited the high antioxidant activities in the DPPH (IC_50_ values: 59.40 and 31.02 µg/mL, respectively), ABTS (IC_50_ values: 80.36 and 62.22 µg/mL, respectively), and superoxide radical scavenging assay (IC_50_ values: 107.47 and 80.00 µg/mL, respectively).

**Conclusions:**

The method combines the advantages of generality, rapidity, simplicity, and is useful for the mass production of walnut peptides.

**Graphical Abstract:**
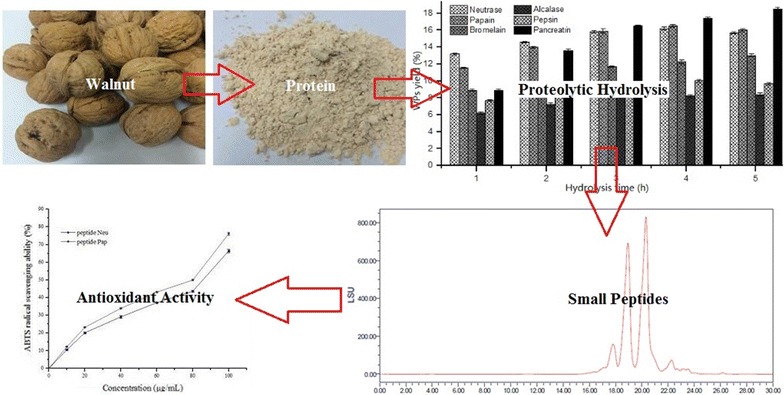
Preparation of antioxidant peptides from walnut (Juglans regia L.) protein hydrolysates

## Background

Oxidative stress has been suggested to be a contributory factor in development and complication of diabetes [1–3]. Antioxidants have been proven to be benefit human health because they may protect the body against molecules known as reactive oxygen species, which can attack membrane lipids, protein and DNA [4, 5]. Reactive oxygen species are atoms, molecules, or ions with unpaired electrons or open-shell configurations, such as hydroxyl radical (^·^OH), superoxide anion radical (O_2_^·−^) [6, 7]. And their formation has been associated with many human diseases, such as heart disease [8], stroke [9], arteriosclerosis [10], diabetes [11], cancers [12], Alzheimer’s disease [13], and major disorders. Therefore, it is very important to inhibit the formation of the excessive amounts of free radicals in food products and the living body. Synthetic antioxidants, such as butylated hydroxyanisole (BHA) and butylated hydroxytoluene (BHT) may be added to food products to retard oxidation reactions [14, 15]. These synthetic antioxidants show stronger antioxidant activities than those of natural antioxidants, such as α-tocopherol and ascorbic acid. However, the use of these chemical compounds has begun to be restricted, because of their induction of DNA damage and their toxicity [16]. Thus, there has been a great deal of interest in finding new antioxidants from natural sources to replace synthetic antioxidants for use in food. In the recent years, many studies have reported that hydrolyzed proteins (peptides) from various animal and plant sources possess antioxidant activity [17–19]. Antioxidant activity of these peptides was enhanced by the presence of hydrophobic amino acids (proline and leucine) in the *N*-terminus [20], and hydrophobic amino acids can increase the accessibility of the antioxidant peptides to hydrophobic cellular targets such as the polyunsaturated chain of fatty acids of biological membranes [21].

Walnut (*Juglans regia* L.), that belongs to the Juglandaceae family, is one of the nuts commonly found in Chinese diets [22, 23]. It is native to the mountain ranges of Central Asia, extending from Xinjiang province of western China [24–27]. Walnut is received increasing interest as nutraceutics mainly due to the fact that their regular consumption has been reported to reduce the risk of coronary heart disease [28]. In addition, many biological activities for walnut have been reported, such as antiatherogenic, anti-inflammatory and antimutagenic properties [29–31], and antioxidant activities [32, 33]. The health benefits of walnut are usually attributed to their chemical composition. Numerous benefit compounds can be found in walnut. For example, it contains polyphenols [34], flavones [35], polysaccharides [36], aminophenols [37], minerals [38], and so on. Moreover, each ounce of walnuts offers about 17 g of fatty acid and contains about 7 g of protein. Therefore, it is considered a good source of edible oil and proteins. Recently, the use of natural protein hydrolysates has been the subject of several research works, because of their antioxidant potential [39]. Researchers had purified peptides from walnut protein hydrolysates using gel chromatography, and these peptides exhibited the highest antioxidant activities and had angiotensin I-converting enzyme (ACE) inhibitor activity [40, 41]. Every method had its own advantages and disadvantages, so all of these led to our interesting in investigating a large-scale production suitable for walnut peptides.

In the present work, we developed a facile and reproducible preparation of antioxidant peptides from walnut protein hydrolysates. Furthermore, the antioxidant effects of walnut peptides against different free radicals were investigated.

## Results and discussion

### Preparation of WPIs by continuous countercurrent extraction (CCCE) process

CCCE of soluble from biomass materials (such as pulp, sugarcane, fruits, seeds, and pretreated lignocellulose) can be accomplished in a variety of commercial equipment [42]. Nowadays, CCCE process is commonly used for large-scale single product plants like in oilseed industry. The process is a simple and efficient continuous extraction, with respect to yield, energy efficiency and level of sanitation [43]. Therefore, our focus is on CCCE process used in the food-processing industry because these systems are most effective in reducing water requirements.

In the present work, we obtained walnut protein isolates (WPIs) by using CCCE process, and normal process was also used. The comparison of the two methods, CCCE process versus normal process, is summarized in Table 1.

**Table 1:** A comparison of CCCE and normal processes for WPIs extraction

Method	Walnut flour (g)	Water required (mL)	Protein yield (%)	Protein purity (%)
CCCE process	600	9000	30.2	82.5
Normal process	600	18,000	31.0	81.8

As shown in Table 1, both methods were able to extract WPIs efficiently, and the yields and purity for proteins extracted from walnuts were comparable. The protein yield and purity for CCCE process were 30.2 and 82.5 %, respectively, while for normal process were 31.0 and 81.8 %, respectively. However, the volume of water required for normal process was one time more than that for CCCE process. Thus, we did not need so much time to concentrate the protein solutions for CCCE process, which led to energy savings. These findings indicated that CCCE process could reduce production costs greatly, and it was available for WPIs extraction.

### Proteolytic hydrolysis

**Fig. 1:**
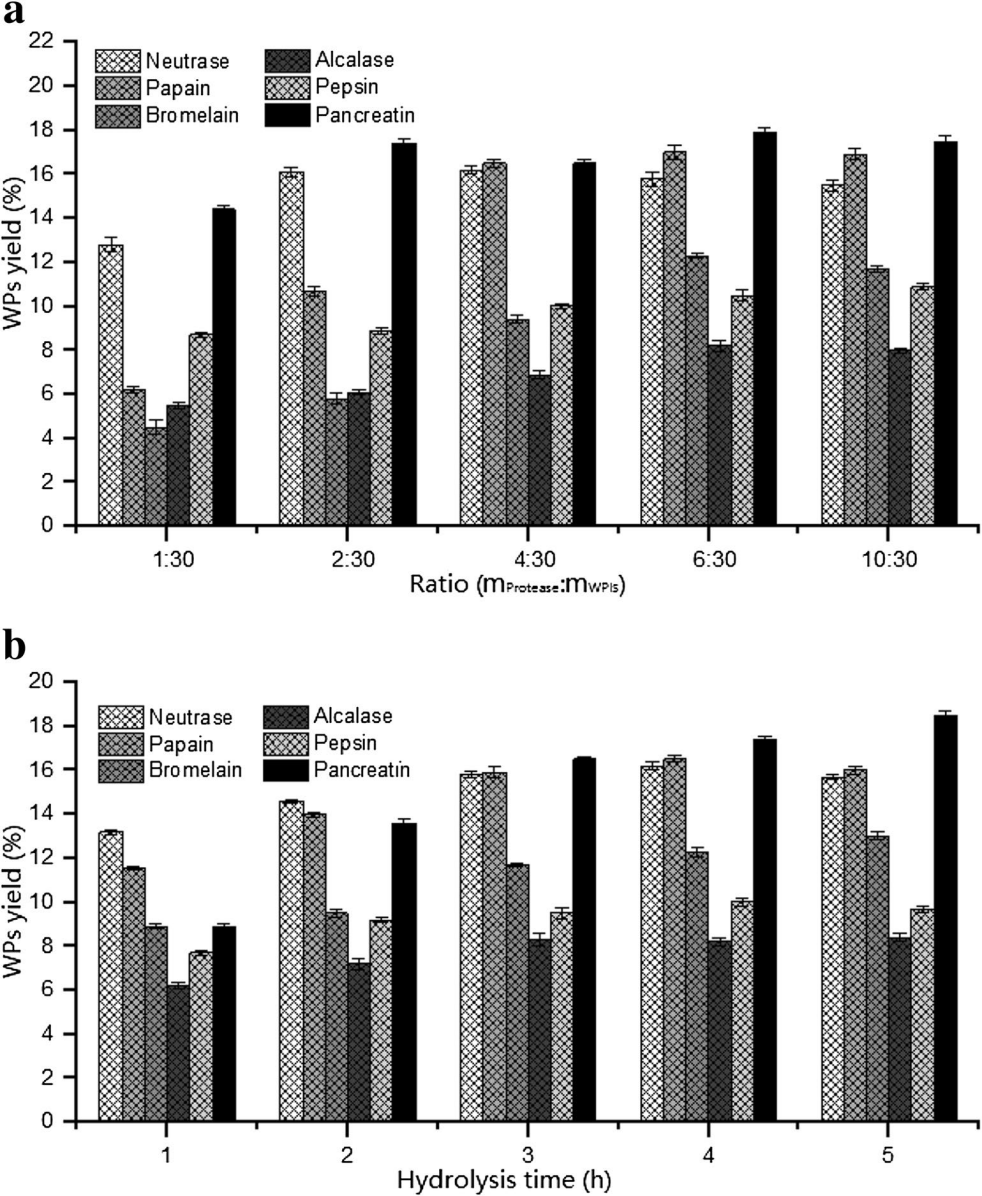
The WPs yields affected by hydrolysis time (**a**) and the protease preparation-to-WP ratios on a weight basis (**b**). All the results are triplicates of mean ± SD

To determine whether the proteases were related to the yields, purity, and activity of peptides, WPIs were separately hydrolyzed by various proteases including neutrase, papain, bromelain, alcalase, pepsin, and pancreatin. Based on the assessment of peptide yields, we studied hydrolysis time and the protease preparation-to-WP ratios on a weight basis (Fig. 1), and the optimum conditions for enzymatic hydrolysis are summarized in Table 2.

**Table 2:** The optimum conditions for enzymatic hydrolysis

Protease	Temp. (^o^C)	pH	Hydrolysis time (h)	Ratio (m_protease_:m_WPIs_)
Neutrase	50	7	4	1:30
Papain	50	7	4	2:30
Bromelain	50	7	4	3:30
Alcalase	50	8	4	3:30
Pepsin	37	2	4	3:30
Pancreatin	50	8	4	2:30

### Purification of peptides from WPHs

Many studies showed that the biological activity of peptides are related to their molecular weight (MW) [44]. Small-size peptides often present an intense biological activity [45]. Therefore, it seems interesting to select purified fractions of peptides of close MW in order to better target their action. Recently, ultrafiltration with high molecular weight cut-off (MWCO) can be used for the separation between peptides and non-hydrolyzed proteins [46].

In the present work, WPIs were separately hydrolyzed with neutrase, papain, bromelain, alcalase, pepsin, pancreatin at optimal conditions. The residue of walnut protein hydrolysates (WPHs) was removed completely using a PVDF flat microporous membrane with MWCO of 200 kDa. An ultrafiltration membrane with MWCO of 2 kDa was used to separate the WPHs into two fractions, WPH-a (MW < 2 kDa) and WPH-b (MW > 2 kDa). WPH-a was collected and concentrated. And then it was spray-dried.

As we know, trichloroacetic acid (TCA) is one of the commonly used protein precipitants [47]. Low molecular weight peptides (small acid-soluble proteins, SASPs) including free amino acids can be dissolved in 15 % TCA (GB 22492-2008 standard in China). The contents of SASPs and free amino acids can be determined by Kieldahl method and using an amino acid analyzer, respectively. The peptide content was calculated according the following formula:

$$X = X_{1} - X_{2}$$

where *X* was the content of peptides (%), *X*_1_ was the content of SASPs (%), and *X*_2_ was the content of free amino acids (%).

Thus, the crude proteins (CP) and ASPs contents of walnut peptides (WPs) were determined by Kieldahl method, and the contents of free amino acids were detected using an amino acid analyzer. The results are summarized in Tables 3 and 4.

**Table 3:** The yields and purity of peptides prepared by six proteases

Protease	WPs yield (%)	CP content (%)	ASPs content (%)	FAA content (%)	WPs purity (%)
Neutrase	16.21	90.55	87.16	6.14	81.02
Papain	16.54	92.47	91.99	7.56	84.43
Bromelain	12.36	83.81	77.41	18.20	59.21
Alcalase	8.25	85.89	75.33	12.40	62.93
Pepsin	10.04	84.00	59.33	1.63	57.70
Pancreatin	17.41	80.15	76.90	31.70	45.24

**Table 4:** Free amino acid contents of peptides prepared by six proteases

Free amino acids	Amino acid contents of peptides prepared by six proteases (%)					
	Neutrase	Papain	Bromelain	Alcalase	Pepsin	Pancreatin
Asp	0.05	0.11	0.12	0.11	0.03	0.62
Thr	0.07	0.12	0.63	0.21	0.01	0.94
Ser	0.25	0.26	1.10	0.63	0.02	0.86
Glu	0.27	0.19	2.04	1.09	0.05	1.58
Pro	ND	0.02	0.07	ND	ND	ND
Gly	0.08	0.55	0.96	0.26	ND	0.44
Ala	0.40	0.30	1.25	1.23	0.03	1.30
Val	0.23	0.16	0.49	0.49	0.07	1.70
Met	0.04	0.06	0.61	0.39	0.01	0.22
Ile	0.22	0.09	0.51	0.24	ND	1.58
Leu	0.63	0.63	2.72	0.99	0.06	4.49
Tyr	0.53	0.79	1.44	0.94	0.34	3.68
Phe	1.79	2.09	2.83	3.42	0.74	4.40
His	0.16	0.18	0.48	0.32	0.07	0.72
Lys	ND	0.39	1.02	1.02	ND	1.98
Arg	0.99	1.62	1.92	1.06	0.02	7.16
Total	6.14	7.56	18.2	12.4	1.63	31.7

As shown in Table 3, the yields of peptides obtained from WPHs by the six proteases were ranging from 8 to 18 %. The three proteases (neutrase, papain, and pancreatin) seemed to be much more efficient. Namely, their effectiveness was better than that of others, with peptide yields of 16.2, 16.5, and 17.4 %, respectively. Also, the WPIs were difficult to be hydrolyzed by alcalase, with yield not exceeding 10 %. CP contents of the six peptides were no less than 80 %, which indicated that the six proteases had no obvious impact on protein content (about 80 %). The peptide produced by pepsin (WPs-Pep) had low ASPs content (59.33 %), which revealed that walnut proteins were difficult to be broken down into small-size peptides by pepsin. In contrast, the ASAPs contents of peptides prepared by neutrase (WPs-Neu) and papain (WPs-Pap) were 87.16 and 91.99 %, respectively. The data suggested that the two proteases were very efficient.

The total contents of free amino acids of the two peptides were 6.14 and 7.56 %, respectively. Thus, their purity was very good: 81.0 and 84.4 %, respectively. However, the total contents of free amino acids in other peptides prepared by bromelain (WPs-Bro), alcalase (WPs-Alc), and pancreatin (WPs-Pan) were exceeding 15 %, which led to low peptide contents. Table 4 shows the contents of free amino acids in the six WPs. Sixteen free amino acids (Asp, Thr, Ser, Glu, Pro, Gly Ala, Val, Met, Ile, Leu, Tyr, Phe, His, Lys, Arg) were found in WPs-Pap and Bro. Pro was not found in WPs-Neu, Alc, Pep, and Pan. Lys was not detected in WPs-Neu and Pep. Ile and Gly also were not found in WPs-Pep. Phe and Arg contents in WPs-Neu, Pap, Bro, Alc and Pan were very high. The contents of Phe in WPs-Neu and Pap were 1.79 and 2.09 %, respectively, while for Arg, the contents were 0.99 and 1.62 %, respectively. This disparity may be due to the different proteases. Likewise, the kind of protease had a significant impact on the contents of amino acids.

All in all, the two types of proteases (neutrase and papain) could hydrolyze WPIs efficiently, which should be selected for further use to prepare WPs. The yield and purity of WPs were 16 and 81 % at least, respectively. This method provided a simple and convenient route for the large-scale preparation of WPs, and it showed huge in practical applications.

### Molecular weight distribution of WPs

**Fig. 2:**
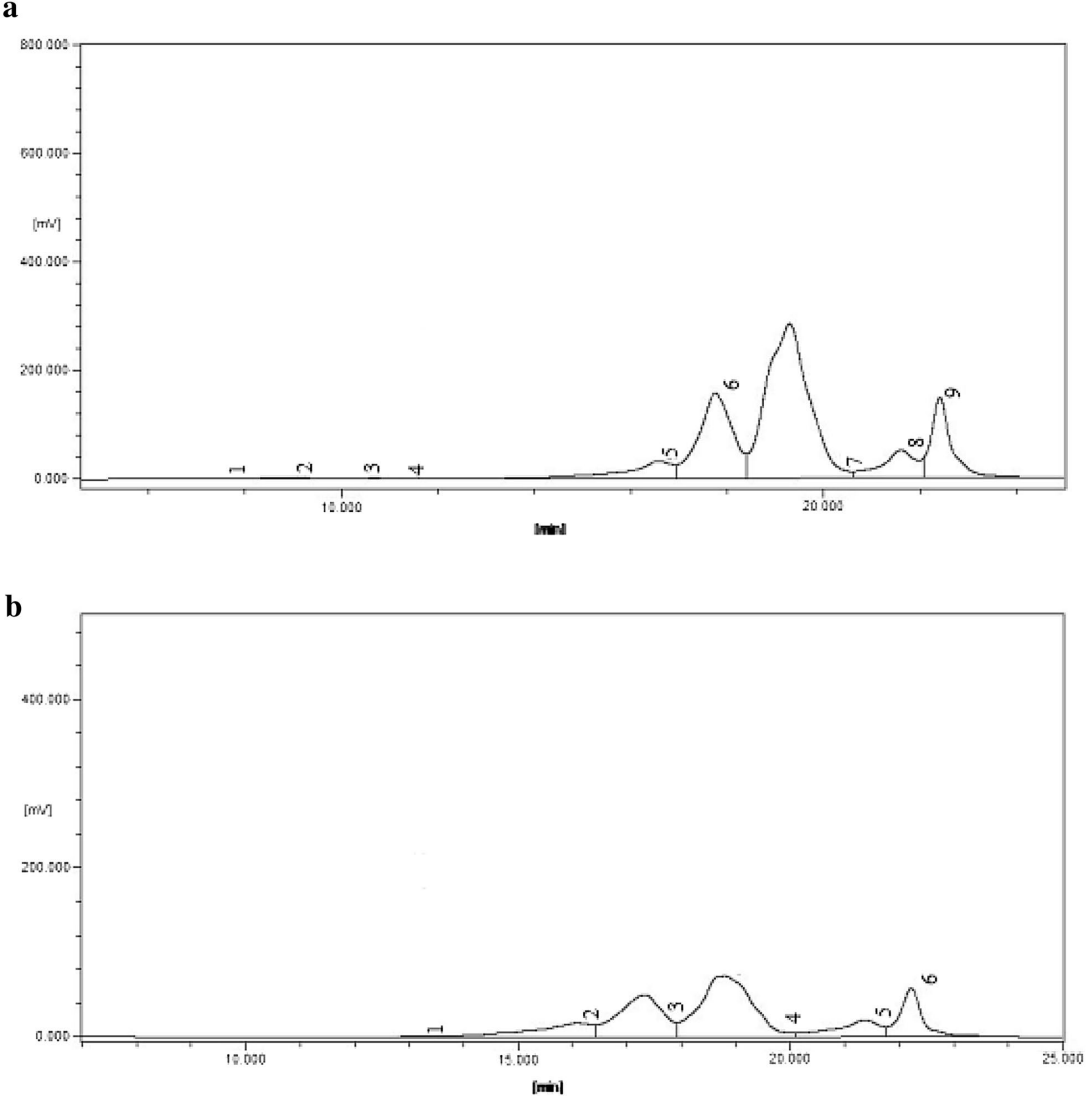
Size exclusion chromatography of WPs-Neu (**a**) and Pap (**b**) on TSK-gel 2000 SWXL column (7.8 × 300 mm) eluted in 20 % acetonitrile with 0.1 % TFA at a flow rate of 0.5 mL/min

In this study, WPs-Neu and Pap were selected to analyze molecular weight distributions. To study the molecular weight distributions of peptides, sized exclusion chromatography with an HPLC system was used (Fig. 2). And the results are summarized in Table 5.

**Table 5:** Apparent molecular weight (Mw) values of peptides

Retention time (min)	Mw	Area (%)	
		WPs-Neu	WPs-Pap
11.31	13,500	0.08	0.64
16.58	1330	6.39	11.25
17.76	492	22.02	23.97
19.30	221	50.37	40.95
21.61	70	7.50	9.31
22.42	40	12.74	13.89

As shown in Table 5, The chromatographic data indicated both peptides were nearly all composed of lower molecular weight peptides. Both peptides had high quantities (99.10 and 99.37 %) of peptides below 1500 Da with major molecular weight located at 200–1500 Da (60 % at least). The results obtained indicated that enzymatic hydrolysis followed by membrane separation was effective in producing walnut peptides and in removing large peptides or undigested proteins.

As far as we know, hydrolytic process of proteins by proteases could generate molecules ranging from individual amino acids to peptides of various sizes and peptide length was thought to be closely related to biological activities. It was reported that low molecular weight peptides had high solubility, low viscosity, and low allergenicity [45, 48]. These peptides are better candidates than longer peptides to play a physiological role in vivo as they are less susceptible to undergo gastrointestinal hydrolysis [49]. And short peptides may be absorbed easily and transported from the intestinal lumen into the blood circulation more efficiently than either amino acids or intact proteins [50]. Additionally, many studies have shown that peptides with low molecular weights exhibit potent ACE inhibitory activity [51]. Thus, the high low molecular weight peptide content could be expected to be beneficial.

### Antioxidant activity

To determine whether WPs could exert significant antioxidant activity, WPs-Neu and Pap were selected to evaluated using 2,2-diphenyl-1-picrylhydrazyl (DPPH), 2,2′-azino-bis (3-ethylbenzothiazoline-6-sulphonic acid (ABTS), and superoxide radical radical scavenging capacity assay.

#### DPPH scavenging activity of peptides

**Fig. 3:**
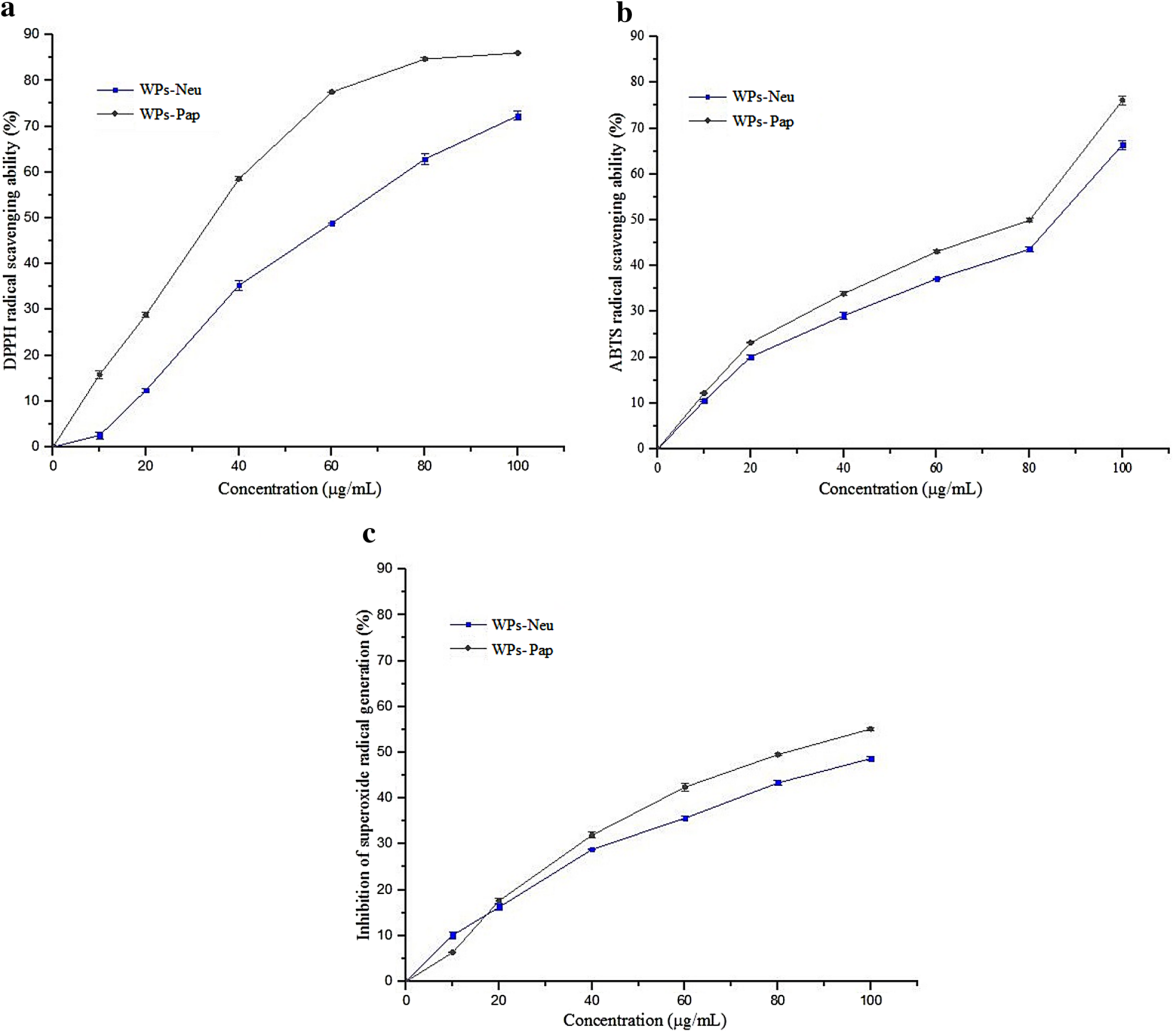
In vitro antioxidant activities of WPs-Neu and Pap in different concentrations. **a** DPPH radical scavenging ability; **b** ABTS radical scavenging ability; **c** superoxide radical scavenging activity. All the results are triplicates of mean ± SD

DPPH radical scavenging assay has been widely used to evaluate the antioxidant capacity [52], which is stable due to its resonance stability and special blockade of benzene rings [53]. The purple chromogen radical DPPH is reduced by antioxidant compounds to the corresponding pale yellow hydrazine [54]. The activities of WPs-Neu and Pap were evaluated, with gallic acid (GA) as positive control. As shown in Fig. 3a, the scavenging activities of DPPH radical by the two WPs increased with increasing concentration. At a concentration of 100 µg/mL, the activities of WPs-Neu and Pap were 72.29 and 86.02 %, respectively. And the IC_50_ values of the two pepties were 59.40 and 31.02 µg/mL, respectively, higher than that of GA (IC_50_: 11.25 µg/mL). It should be noted that the scavenging activity of WPs-Pap was higher than that of WPs-Neu. Therefore, the results indicated that WPs-Pap had strong DPPH radical scavenging activity.

#### ABTS radical scavenging activity of peptides

The peroxidase substrate ABTS, forming a relatively stable radical (ABTS^·^) upon one-electron oxidation, has become a popular substrate for estimation of total antioxidant capacity [55]. ABTS radical assay is an excellent tool for determining the antioxidative activity, in which the radical is quenched to form ABTS radical complex [56]. Meanwhile, it is more sensitive to determine antioxidative capacities of protein hydrolysates samples, because it can determine their capacities at lower inhibition concentrations. ABTS radical scavenging properties of WPs-Neu and Pap are present in Fig. 3b. With increasing concentration, the two peptides showed increased ABTS radical scavenging activities, and their scavenging rates were 66.41 and 76.14 %, respectively. The IC_50_ value of WPs-Neu was 80.36 µg/mL, while for WPs-Pap, the IC_50_ value was 62.22 µg/mL. These values suggested that WPs-Pap had higher scavenging activity than that of WPs-Neu, consistent with the results for DPPH radical scavenging assay.

#### Superoxide radical scavenging activity of peptides

The superoxide anion radical is the most common free radical generated in vivo. Superoxide anion, derived from dissolved oxygen by a phenazine methosulphate (PMS)-NADH coupling reaction, reduces nitroblue tetrazolium (NBT) [57]. The decrease in absorbance at 560 nm in the presence of antioxidants indicates the consumption of superoxide anions. Figure 3c shows percentage inhibiton of superoxide anion radical generation for different amounts of WPs-Neu, compared with the same concentration of WPs-Pap. It can be seen from Fig. 3c that the two peptides showed dose dependent activity. The scavenging ratios of WPs-Neu and Pap at 100 µg/mL were 48.66 and 55.13 %, respectively, and the IC_50_ values were 107.47 and 80.00 µg/mL, respectively. These results indicated WPs-Pap is a good scavenger of the superoxide radical.

## Conclusions

In this study, we developed a simple and convenient method for the large-scale preparation of WPs. Walnut proteins were obtained using CCCE process, and separately hydrolyzed with neutrase, papain, bromelain, alcalase, pepsin, pancreatin at optimal conditions. The peptides were further purified from protein hydrolysates through using an ultrafiltration membrane with MWCO of 2 kDa. Our data indicated that two types of proteases (neutrase and papain) could hydrolyze WPIs efficiently, which should be selected for further use to prepare WPs. The yield and purity of WPs prepared using the two proteases were 16 and 81 % at least, respectively, and the peptides had high quantities (99 % at least) of peptides below 1500 Da with major molecular weight located at 200–1500 Da. In addition, the antioxidant effects of the two walnut peptides were tested using DPPH, ABTS and superoxide radical scavenging capacity assays. The results revealed that both possessed excellent antioxidant activities. Therefore, this study may be of high interest for the food industry, and the method showed huge in practical applications.

## Experimental

### Reagents and chemicals

Walnuts (*Juglans regia* L.) were purchased from a local market in Xinjiang province, China. Neutrase (powder, ≥600 units/mg solid) and papain (powder, ≥1000 units/mg solid) were procured from Guangxi Pangbo Biothech Co., Ltd. Reagents of analytical grade (sodium hydroxide, hydrochloric acid, trifluoroacetic acid, trichloroacetic acid) were obtained from Sinopharm Chemical Regent Co., Ltd., and used without further purification unless otherwise noted. Acetonitrile (HPLC grade) was obtained from Merck Millipore Corp. Ultrapure water from a Milli-Q water purification system was filtered through a 0.22 µm membrane filter before use.

### Preparation of WPIs

Walnut kernels were defatted using cold-pressing technology. The WPIs were obtained using CCCE process.

1. The defatted flour **A** (200 g) was dispersed in 3000 mL of sodium hydroxide solution (pH 9.5), and extracted at 40 °C. After being stirred for 1 h, the mixture was centrifuged at 1500×*g* for 10 min to get residue **A** and supernatant **A**. The residue **A** was extracted with sodium hydroxide solution again, and then was centrifuged to yield supernatant **B**.
2. The defatted flour **B** (200 g) was dispersed in supernatant **A**, and the pH of the mixture was adjusted to 9.5. After being stirred for 1 h at 40 °C. The mixture was centrifuged at 1500×*g* for 10 min to get residue **B** and supernatant **C**. The residue **B** was extracted with sodium hydroxide solution again, and then was centrifuged to yield supernatant **D**.
3. The defatted flour **C** (200 g) dispersed in supernatant **B** was extracted a second time. Residue **C** and supernatant **E** were obtained by centrifuging the mixture. The residue **C** was poured into the **s**upernatant **D**, and was extracted again. The mixture was centrifuged to obtained supernatant **F**. At last, the supernatant **C**, **E,** and **F** were combined, and its pH was adjusted to 4.5. After 30 min, the supernatant was discarded to get WPIs.

### Preparation of WPHs

WPIs were dissolved in about 3000 mL of water at a total volume of 5000 mL to obtain a protein concentration of 3 %, and hydrolyzed with neutrase (5 g) or papain (10 g). Temperature and pH conditions were adjusted to 50 °C and 7.0, respectively. Agitation was maintained at a constant of 300 rpm. The pH was kept constant using 0.5 M sodium hydroxide solution. After 5 h, neutrase or papain was heat-deactivated at 95 °C for 10 min in a water bath. The mixture was centrifuged at 1500×*g* for 20 min at 20 °C, and residue was discarded to obtain WPHs.

### Purification of WPs

The residue was further removed from WPHs using a PVDF flat microporous membrane with MWCO of 200 kDa. Then, WPHs were further purified through an ultrafiltration membrane with MWCO of 2 kDa, and concentrated using evaporator under vacuum at 60 °C to afford about 1000 mL of WPHs, which were spray-dried to obtain WPs.

### Determination of walnut peptide content

#### Determination of SASPs content

1 g of WPs was weighed and dispersed in a 50 mL volumetric flask with a moderate amount of 15 % TCA under ultrasonic conditions, and then diluted to scale. The dispersions were separated into supernatant and precipitate with a suction filter [58]. The content of supernatant was then determined using Kjeldahl method, which was performed as previously described [59].

#### Determination of free amino acids content

The free amino acid analysis was carried out according to the method described by Zhang et al. [60].

### Determination of molecular weight distribution

The molecular weight distribution was determined by gel permeation chromatography on a TSKgel G2000SWXL column (7.8 mm × 300 mm i.d., 5 µm) with a HPLC system according to the method of Gu et al. [61]. HPLC was carried out with the mobile phase (20 % acetonitrile with 0.1 % TFA, v/v) used at a flow rate of 0.5 ml/min and monitored at 220 nm at 27 °C. The standards used were tripeptide GGG (Mr 189), tetrapeptide GGTA (Mr 451), bacitracin (Mr 1422), and Insulin (Mr 5777) (Sigma Chemical Co., USA).

### DPPH radical scavenging assay

All tested samples were dissolved in ethanol. 100 µL of DPPH in ethanol was added into a 96-well plate, and was mixed with the test samples (100 µL) at different concentrations. After shaken for 60 s in microplate reader, it was left in the dark at 37 °C for 30 min. The absorbance was then measured at 515 nm with a microplate reader (BIO-RAD, model 680) [62]. All experiments were carried out in triplicate. Ethanol was used as the blank control and vitamin C served as positive control. The DPPH radical scavenging activity were calculated according to the following formula.

$$\% \,{\text{DPPH}}\,{\text{Scavenging}}\,{\text{activity}} = (A_{\text{blank}} - \, A_{\text{sample}} )/A_{\text{blank}} \times 100$$

### ABTS radical scavenging assay

ABTS and potassium persulfate were dissolved in distilled water to a final concentration of 7 and 2.6 mmol/L, respectively, and mixed. The mixture allowed to stand in the dark at room temperature for 12 h before use. It was then diluted by mixing 1 mL ABTS solution with 60 mL of phosphate buffered saline (PBS) to obtain an absorbance of about 1.00 at 734 nm using a spectrophotometer. All tested samples were dissolved in PBS. 5 mL of fresh ABTS solution was mixed with 500 µL of tested samples for 2 h in a dark condition. The absorbance was then measured at 734 nm with a spectrophotometer [63]. All experiments were carried out in triplicate. PBS was used as the blank control and vitamin C served as positive control. The ABTS radical scavenging activity were calculated according to the following formula.

$$\% \,{\text{ABTS}}\,{\text{scavenging}}\,{\text{activity}} = (A_{\text{blank}} - \, A_{\text{sample}} )/A_{\text{blank}} \times 100$$

### Superoxide radical scavenging activity

All tested samples were dissolved in Tris–HCl (16 mmol/L, pH 8.0). The superoxide radicals were generated in 5 mL of reaction mixture containing 1 mL of NBT (300 µmol/L) solution, 1 mL of NADH (468 µmol/L) solution and 3 mL of sample solution were mixed. The reaction started by adding 1 mL of phenazine methosulphate (PMS) solution (60 µmol/L) to the mixture. After 5 min, the absorbance was then measured at 558 nm with a spectrophotometer [64]. Tris–HCl was used as the blank control and vitamin C served as positive control. All experiments were carried out in triplicate. The percentage inhibition of superoxide anion generation was calculated using the following formula.

$$\% \,{\text{superoxide}}\,{\text{radical}}\,{\text{scavenging}}\,{\text{activity}} = (A_{\text{blank}} - \, A_{\text{sample}} )/A_{\text{blank}} \times 100$$

### Statistical analysis

All statistical analyses were performed using SPSS 10.0, and the data were analyzed using one-way ANOVA. The mean separations were performed using the least significant difference method. Each experiment was performed in triplicate, and all experiments were run thrice and yielded similar results. Measurements from all the replicates were combined, and the treatment effects were analyzed.

## Abbreviations

ABTS: 2,2′-azino-bis(3-ethylbenzothiazoline-6-sulphonic acid
ACE: angiotensin I-converting enzyme
BHA: butylated hydroxyanisole
BHT: butylated hydroxytoluene
CCCE: continuous countercurrent extraction
DPPH: 2,2-diphenyl-1-picrylhydrazyl
GA: gallic acid
MWCO: molecular weight cutoff
NBT: nitroblue tetrazolium
PMS: phenazine methosulphate
SASPs: small acid-soluble proteins
TCA: trichloroacetic acid
WPs: walnut peptides
WPHs: walnut protein hydrolysates
WPIs: walnut protein isolates
